# Implants as a treatment alternative in children with multiple agnesia: Systematic review and meta-analysis

**DOI:** 10.4317/jced.60168

**Published:** 2023-04-01

**Authors:** Mª Dolores Casaña-Ruiz, Montserrat Català-Pizarro, Carlos Borrás-Aviñó, Mª Filomena Estrela-Sanchís, Carlos Bellot-Arcís, Jose Mª Montiel-Company

**Affiliations:** 1 Estudiante de máster de odontopediatría. Departamento de Estomatología. Facultad de Medicina y Odontología. Universitat de València. Valencia. Spain; 2 Profesora Titular de Odontopediatría. Departamento de Estomatología. Facultad de Medicina y Odontología. Universitat de València. Valencia. Spain; 3 Profesor asociado de la Universitat de València. Departamento de Estomatología. Facultad de Medicina y Odontología. Conceived the ideas. Valencia. Spain; 4 Profesor contratado Doctor. Facultad de Medicina y Odontología. Universitat de València. Valencia. Spain; 5 Titular Departamento de Estomatologia. Facultad de Medicina y Odontología. Universitat de València. Valencia. Spain

## Abstract

**Background:**

The bone growth factor was a conditioning circumstance that limited the use of implants in children and adolescents, which, in cases of anodontia or severe oligodontia, forced pediatric dentists to abandon their use, leaving children with removable prostheses, at an age and in a social context with increasingly functional and esthetic demands. Purpose. The objective is to assess which variables influence the survival of dental implants in pediatric patients with severe agenesis.

**Material and Methods:**

A search was carried out in the Pubmed, Scopus and Web of Science databases, which was completed with a manual search.

**Results:**

The following variables were extracted from the selected studies: author and year, number of patients or cases described, age, gender, number of implants, follow-up time, implanted area, percentage of success or failure, medical and dental history of the patients, type of treatment and study design.

**Conclusions:**

The use of implants as a treatment at an early age has been a controversial issue. Using the appropriate preventive measures, the clinician can offer the child or adolescent a better life quality, esthetics and functionality, until the growth completion period allows for more complex and extensive rehabilitative treatments. A success rate of 89.8% was established for these implants, with no association with follow-up time or type of implant used. The highest survival rates were reported in the anterior mandibular region.

** Key words:**Pediatric dentistry, ectodermal dysplasia, anodontia, oligodontia and dental implant.

## Introduction

Agenesis is a congenital anomaly mainly of genetic origin that results in the lack of formation or development of the dental germs, which determines the absence, from the first years of life, of one or more teeth in the mouth.1 

The traditional treatment of multiple agenesis is usually carried out in several phases, depending on the age of the patient, using different prosthetic options depending on the number of teeth present in the mouth. When the number of teeth is significant we resort to bonded prostheses; when the number of teeth remaining in the mouth is reduced we place a removable partial prosthesis and when the absence of teeth is total we have no other option but to resort to a complete prosthesis (1).

In adulthood, the regular use of dental implants has made it possible to fully satisfy these requirements linked to the quality of life associated with dental health. In children, because of the impact of growth on the position of implants, these have traditionally been banned (2).

The increased current knowledge on parameters regarding facial development and on the evolution and displacement that an element fixed to a growing jaw, such as an implant, would undergo, means that the clinician can anticipate its behavior. Therefore, we can resort to it as an element that, at an early age, can provide us with the added retention for the prosthesis offering improvements in the functionality, esthetics and life quality of the children treated (2).

The objective is to assess which variables influence the efficacy of dental implants in pediatric patients with severe agenesis.

## Material and Methods

A systematic review of the literature was carried out in accordance with the PRISMA recommendations (PRISMA 2020 (Preferred Reporting Items for Systematic Reviews and Meta-Analyses; The PRISMA 2020 statement: an updated guideline for reporting systematic reviews (3) The review protocol has been registered in PROSPERO (CRD42021231031).

-Search Strategy 

PICO question: Which parametres (O) influence the placement of dental implants (I) in pediatric patients (P) with severe agenesis?

In order to identify the most relevant studies, three different electronic databases were used: Pubmed, Scopus and Web of Science. A manual search was also carried out in Gray Literature, Opengrey. In specific cases, the authors of the articles were contacted by e-mail in order to request additional information. In addition, the references of the resulting studies were examined for potentially eligible studies that did not appear in the preliminary database search. This review was last updated as of December 20, 2021.

The search strategy was designed considering previous studies in the field and their most cited descriptors. The keywords to identify the articles were: #1 “pediatric* dentistr*” or “paediatr* dentistr*” or child* or infant* or bab* or boy* or kid* or preschool* or newborn* AND #2”hypodontia” or “oligodontia” or “anodontia” or “tooth agenes*” or “dental agenes*” or “dysplasia*” or “ectodermal dysplasia*” or “ectodermal defect” or “ectodermal dysplasia* anhidrotic” or “congenital ectodermal defect*” AND #3”mini-implant*” or mini implant* or “miniscrew*” or “microimplant*” or “transitional implant*” or “dental implant*” or “over denture* “ or overdenture* or “surgical dental protheses*” or “orthodontic* therap*” or prosthodontic* or “orthodontic treatment” or “prosthetic dentistry” or “dental prostheses” or “dental prostheses”.

-Selection criteria 

Inclusion and exclusion criteria: Randomized clinical trials (RCTs), longitudinal studies, cohort or case-control studies, both retrospective and prospective, were included. No restrictions were placed on the year of publication or language. Inclusion criteria were the following: human studies, particularly in children up to 17 years and 11 months old (pediatric age), where implants were placed for the treatment of severe agenesis. No exclusion criteria were established.

References identified by the search strategy were exported from each database to the Mendeley reference management software (Elsevier, Amsterdam, The Netherlands) to check for duplicates. Two reviewers (MD-CR and CB-A) independently evaluated the titles and abstracts of all identified articles, and in case of disagreement a third reviewer was consulted. If the abstract did not provide sufficient information to make a decision, the reviewers read the entire article. Subsequently, all articles were read in full and the reasons for rejecting the excluded articles were recorded (Table 1).

**Table 1:**
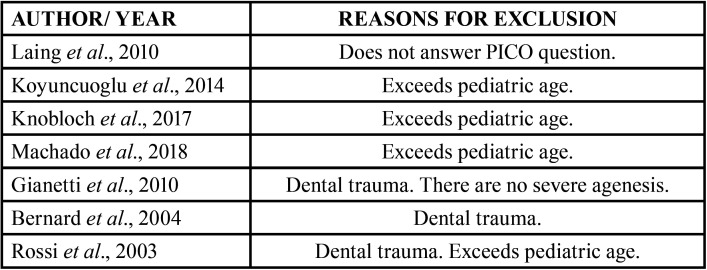
Excluded articles and their reasons.

-Data collection and analysis 

The following variables were extracted from the selected studies: author and year, number of patients or cases described, age, gender, number of implants, follow-up time, implanted area, success rate, medical and dental history of the patients, type of treatment and study design.

Risk of bias/quality assessment in individual studies: The Joanna Briggs Institute checklist (4) was used to assess the quality of case report or case series studies (Table 2), the New Castle-Ottawa scale for cohort studies (5) (Table 3) and AMSTAR 2 for systematic reviews and meta-analyses (6) (Table 4).

**Table 2:**
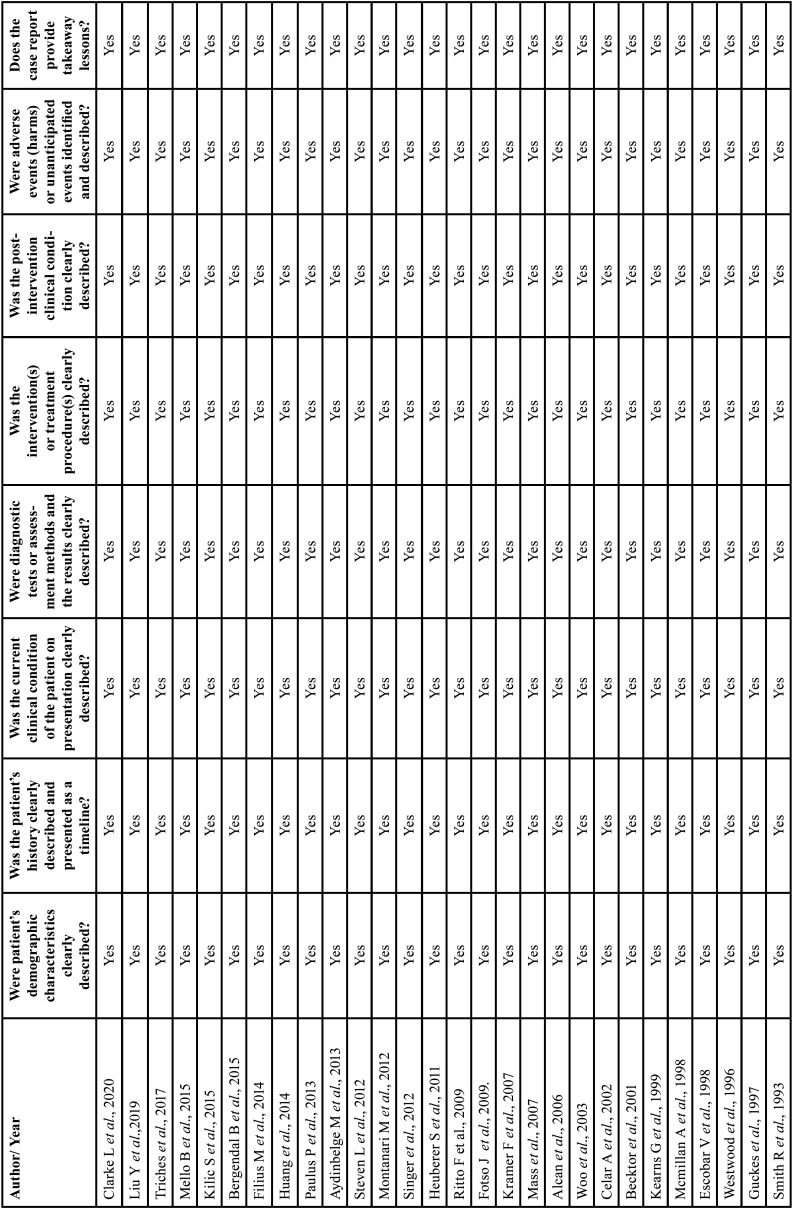
Case Reports.

**Table 3:**
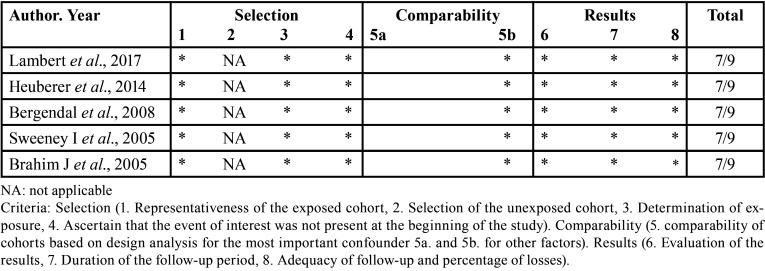
Cohortes studies.

**Table 4:**
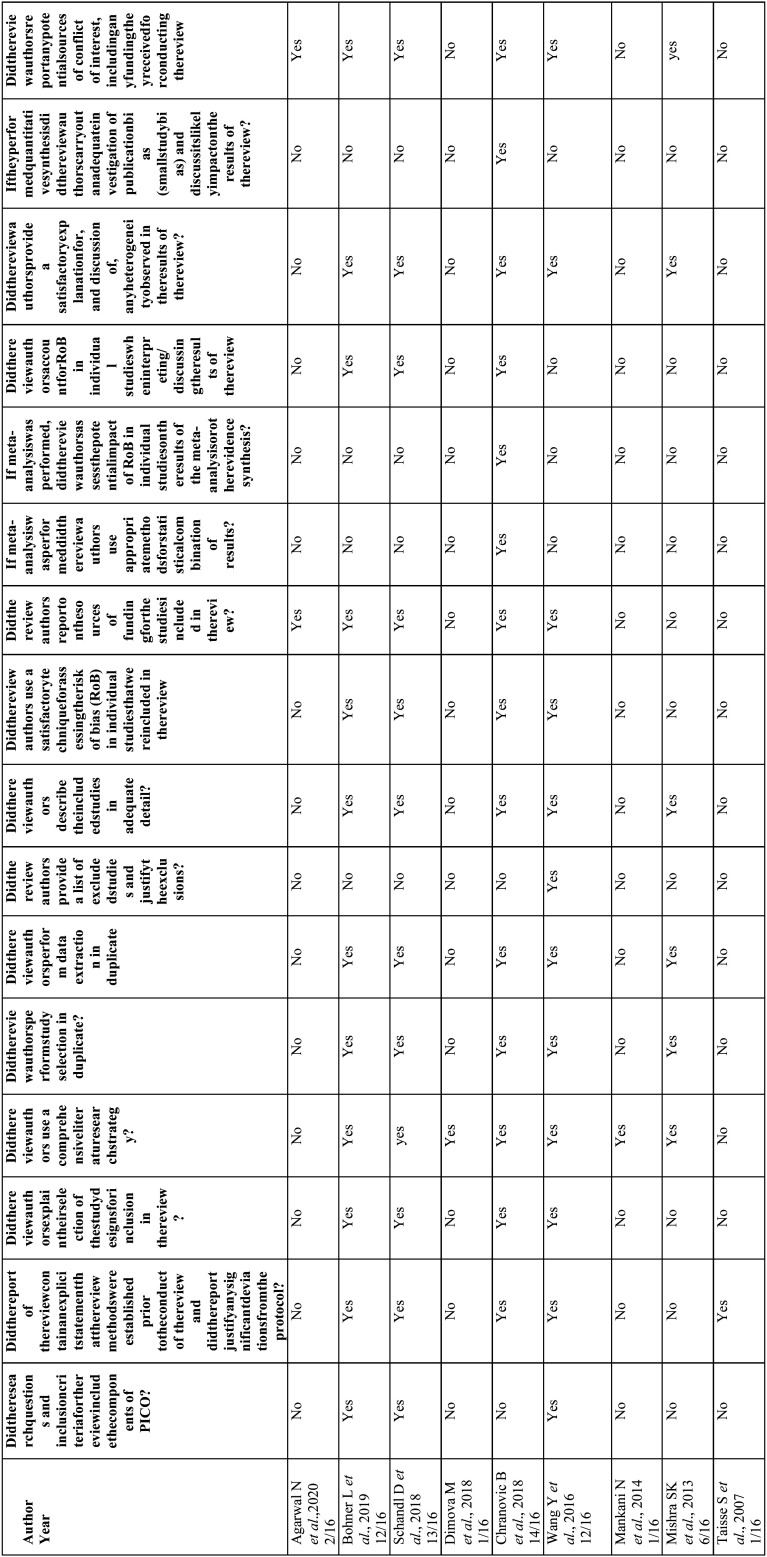
Quality analysis according to AMSTAR 2 scale for systematic reviews and meta-analyses.

Summary measures and approach to synthesis: The mean and confidence interval for follow-up time and implant success rate were collected.

Effect measurement: The percentage of success, calculated as the number of implants remaining in the mouth at the end of the follow-up period, with respect to the total number of implants placed, was expressed as a percentage. By means of the scatter plot or meta-regression it has been possible to evaluate the heterogeneity, registering a tendency towards implant failure, according to a longer follow-up period.

Synthesis methods: For the quantitative analysis or meta-analysis, the studies were combined using a random-effects model. Heterogeneity was assessed using the Q test and the I2 test. A Q test *p-value* of less than 0.1 was considered to indicate heterogeneity.

Risk of bias across studies: Publication bias was assessed using funnel plots, the Trim and Fill method and Egger’s regression Intercept.

## Results

**Figure 1:**
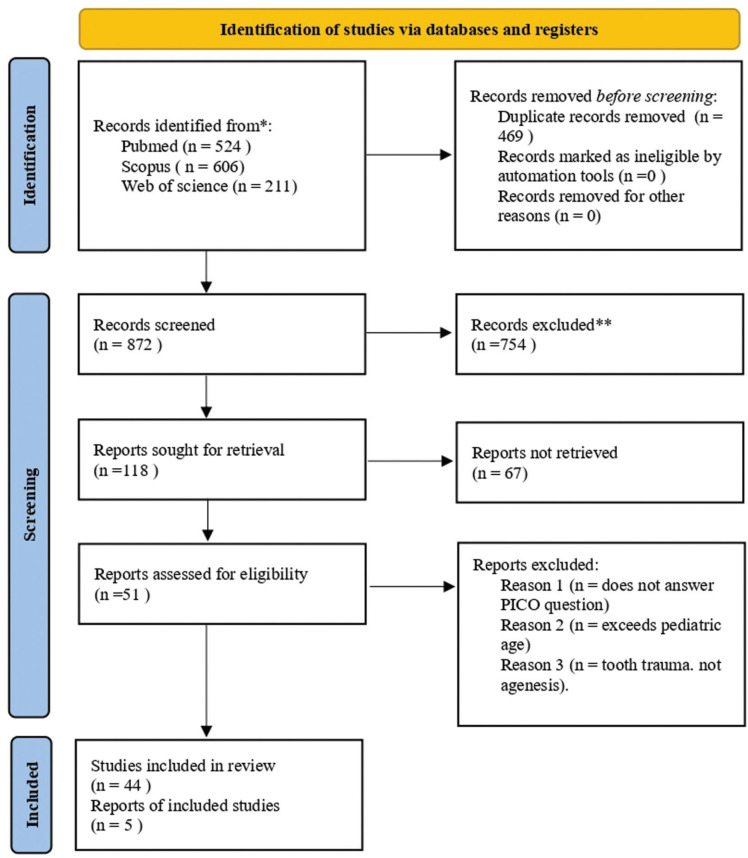
PRISMA 2020 flow diagram for new systematic reviews which included searches of databases and registers only.

Study selection and flow diagram: From the initial electronic search in the 3 databases, 1341 articles were identified: 524 from Pubmed, 606 from Scopus, and 211 from Web of Science. After eliminating duplicate articles, a total of 872 remained. After reading the title and abstract, 118 articles were eliminated, leaving 51 for full-text assessment. After reading the full text, 6 were eliminated for not answering the research question or the inclusion criteria, leaving a total of 45 articles. Only 5 articles were included for the meta-analysis. The PRISMA flow diagram (Fig. 1) provides an overview of the article selection process.

Results of individual studies, meta-analysis and additional analyses: Regarding the number of participants included in the studies obtained, most of them analyze the follow-up of the treatment of a single patient. Brahim 2005 (7), is the one with the highest number of participants, with a total of 35, followed by Bergendal 2008 (8) with 26, Heuberer 2014 (9) with 18 and Lambert 2017 (10) with 12. Similarly, studies such as those by Heuberer 2011 (11) or Kearns 1999 (12) included 6 patients, Filius 2014 (13) or Montanari 2014 (14) included 4 and Clarke 2020 (15), Fotso 2009 (16) or Escobar 1998 (17) only 2.

In reference to the data collected according to the age of the participants, the studies by Bergendal 2015 (18) and Guckes 1997 (19) are the ones that record a smaller sample for the age variable, 3 years. On the contrary, and within the previous established limits, 17 years and 11 months, the works of Brahim 2005 (7), Lambert 2017 (10), Liu 2019 (20), Ritto 2009 (21), Mass 2007 (22), Bernard 2004 (23) and Westwood 1997 (24) are those that collect patients of older ages. On average, it has been observed that the age range with the greatest inclusion for early implant placement is between 8 and 10 years of age.

Regarding gender distribution, the participation of women is notably higher than that of men, except in the studies of Filius 2014 (13) with the inclusion of 3 male patients and Heuberer 2011 (11) with 5 male patients, versus 1 female.

Another variable that has been analyzed is the follow-up period. The study by Bergendal 2015 (18) accompanies the patient for 30 years and authors such as Huang 2014 (25) or Steven 2012 (26), for 20. In contrast, 1 year was the time that Aydinbelge 2013 (27) and his team followed the patient during treatment. Despite the large discrepancy between the aforementioned studies, the mean follow-up time for most cases was of 5 or 6 years with revisions every 6 months.

Regarding the general medical history and clinical history of the patient, practically all the studies presented patients with ectodermal dysplasia with the exception of 3. In the work of Clarke 2020 (15), one of the cases has Hay-Wells syndrome. In the case of Mass 2007 (22) it is the Williams-Beuren syndrome and finally Woo 2003 (28) presents the case of a patient with Papillon-Levefre syndrome. Oral manifestations in most cases are common with severe agenesis or anodontia, hypodontia, microdontia, bone atrophy and/or poor facial musculature.

Continuing with the design of the studies analyzed, 9 of them belong to the group of systematic reviews 30,31,32,33,33,34,35,35,36,37,38, 34 are case series or cohort studies 7,8,9,10,11,12,16,17,21,24,25,25,26,39,40,41,42,43,44,45,46,47,48,49,50,51 and finally Laing 2010 (29) is classified as a cross-sectional-observational study (Table 5- Table 5 cont.-1).

**Table 5:**
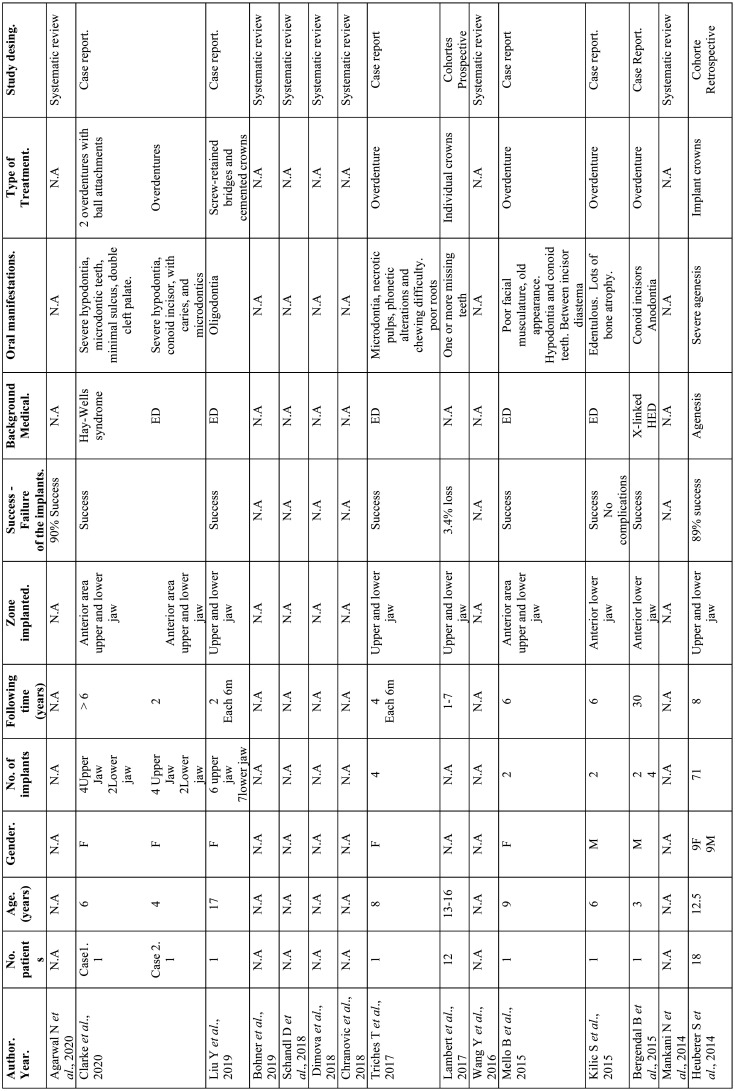
Results of individual studies.

**Table 5 cont.**
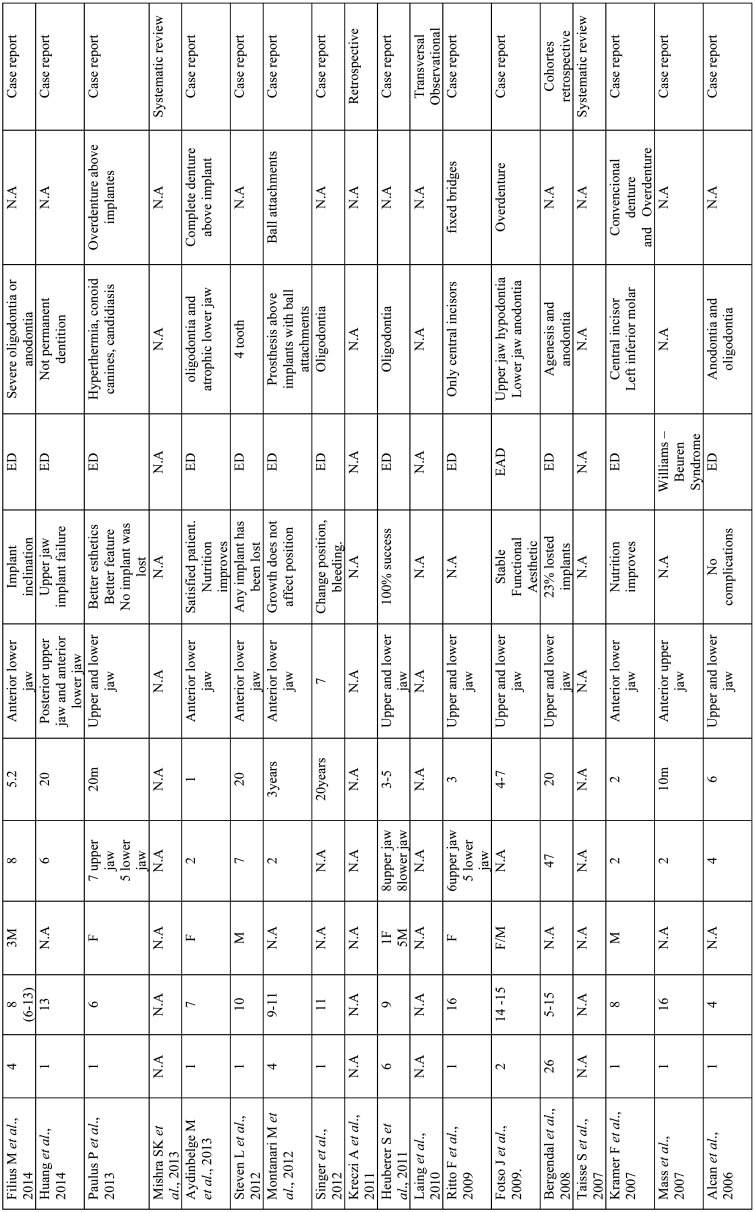
Results of individual studies.

**Table 5 cont.-1:**
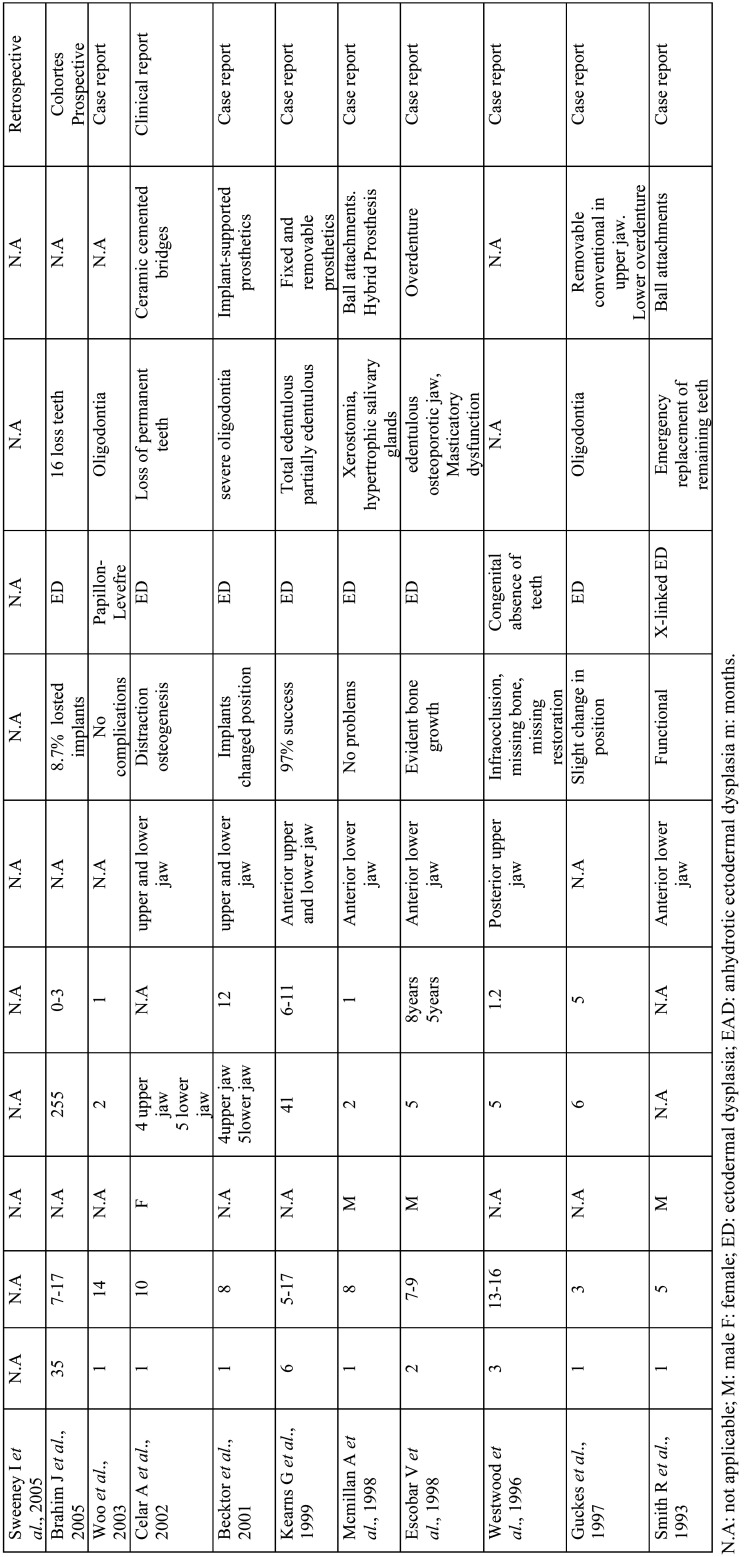
Results of individual studies.

**Figure 2:**
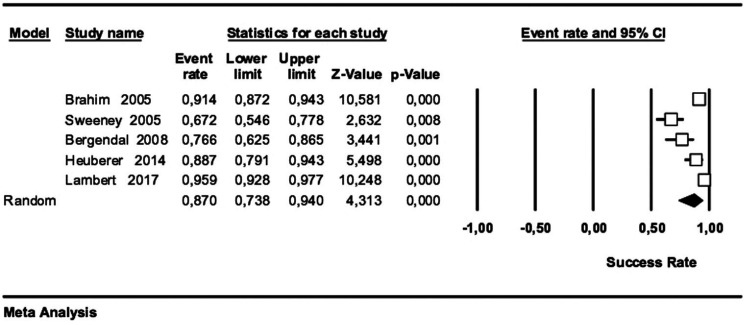
Forest Plot for the estimation of the implant success rate.

Quantitative synthesis: Five cohorts studies were included in the meta-analysis for the estimation of the implant success rate. The studies were combined using a randomized effects model. The success rate was estimated to be 87% with a 95% confidence interval between 73.8 and 94. The meta-analysis showed the existence of heterogeneity (I2: 90.9. Q2: 44.09 *p*<0.001). The Forest plot of the meta-analysis is shown in (Fig. 2).

**Figure 3:**
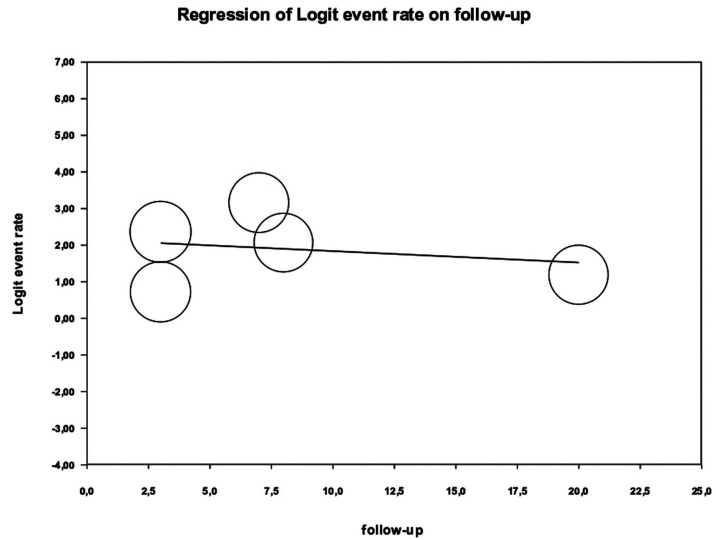
Scatter plot for the influence of the follow-up in the estimation of the implant success percentage.

To study the possible sources of heterogeneity in the meta-analysis, a meta-regression was performed to evaluate the influence of the follow-up time variable in the estimation of the implant success percentage. The results obtained were an intercept coefficient = 2.15 (95% CI: 0.56 and 3.73) and a follow-up time variable = -0.03 (95% CI: -0.19 and 0.12). The meta-regression did not show significance for Q = 0.15, *p* = 0.694. These results indicate that the follow-up period does not significantly influence the percentage of implant success. The results are shown in the scatter plot of (Fig. 3).

**Figure 4:**
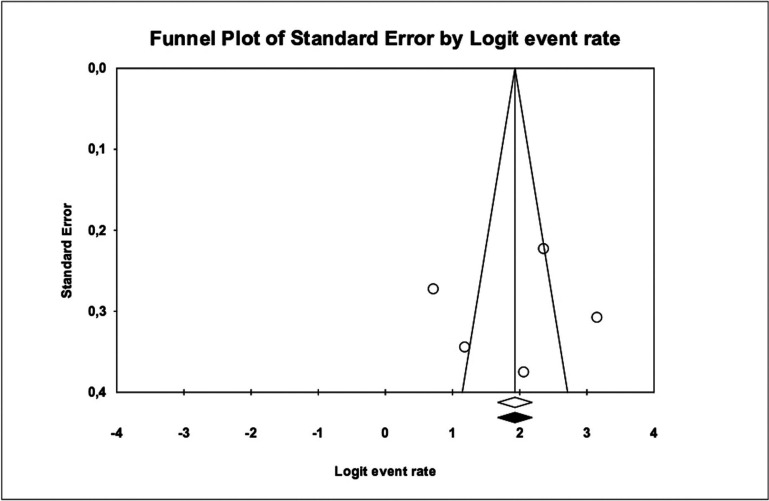
Funnel Plot for the included cohorts studies in the meta-analysis and imputed studies, using the Trim and Fill method.

Publication bias: The Funnel plot presents a symmetrical image to which no new studies could be imputed by the Trim and Fill method. Furthermore, the Egger intercept = -- 1.90 (95% CI: -31.1 and 27.3) including zero and *p* value 0.849. Therefore, it can be considered that there is no publication bias (Fig. 4).

## Discussion

Summary of evidence.

In pediatric patients with multiple agenesis, the functionality for dental implants is controversial. Classically it has been compared to a process similar to dental ankylosis. For this reason they may have a greater number of esthetic and functional complications with lower survival rates than adults (52).

The most frequent causes that would justify the placement of implants at an early age are trauma and agenesis or syndromes with severe hypodontia. The latter are very rare conditions that are usually associated with congenital pathologies such as Down syndrome (trisomy 21) or ectodermal dysplasia, the latter being the most frequent (45).

A better knowledge of the parameters of development and growth, as well as of the multiple existing implantological possibilities, have made it possible to modify the placement timing, so extensively discussed in recent years (2,51). Classically, it has been considered that there is a minimum age for placing implants, 18-21 years in boys and a little earlier in girls, 16-18 years.33 However, Taisse 2017 stated that it is possible to place implants before the aforementioned ages as long as the benefits outweigh the risks.

Moreover, it has been noted that even in adults, there is a slight potential for continued growth, which may have esthetic and functional consequences for the future of the restorations (51).

The most common ages for implant placement were around the age of 8 years old (early adolescents) (13,39). Heuberer 2014 and Liu 2019 were the studies that contained older patients, 17 years of age, in contrast to Bergendal 2015 with his 3-year-old patient. Therefore, there is a wide range of performance, with a remarkable heterogeneity of the treatments.

Due to the great bone atrophy, proper planning is necessary to avoid future complications. Treatments such as bone grafting, osteogenesis distraction and lateralization of the dental nerve at mandibular level are three of the previous surgical procedures that will allow acquiring a notable bone increase 34. In the works of Fotso 2009 and Ritto 2009, grafting from the iliac crest was chosen prior to implant placement. In contrast, in the study by Liu 2019, they performed sinus lift with regeneration and membrane. The previous surgical treatments varied according to the needs of each case.

Regarding the type of implant, Becktor 2021 recommended the use of monoblock mini-implants with reduced diameter. Transitional implants were another option, since they are designed to support temporary prostheses during the time necessary to provide definite solutions (33). However, authors such as Heuberer 2014, resorted to Branemarck MK III Ti (Nobel biocare) since it has an external hexagonal connection, with machined surface, guaranteeing bicortical anchorage in cases of reduced bone density and thus achieving high optimal initial stability.

Prior to placement, special care should be given to the facial pattern and the selected insertion area. The degree or severity of the hypodontia and the possible psychological consequences will also be of vital importance since, if the implants are explanted due to poor planning, there may be negative consequences of greater repercussions (34).

Medina 2017 stated that in brachyfacial patients, the maxillary implants in the anterior sector were more palatalized and more lingualized in the mandible, with a risk of underocclusion in the premolar area. On the other hand, in dolichofacial patients, the mandibular implants could be placed buccaly due to the lingual tendency of the remaining teeth, with a risk of infraocclusion in the anterior area.

It has been observed from the results that in most cases the dominant location was the mandibular anterior region (13,47,58), as opposed to the location of implants in both maxillary and mandibular regions (20,21). Survival rates were consistently higher for implants placed in the mandible (91% to 92%) (55) than for those placed in the maxilla (71% to 86%) (56). In the antero-inferior region, alveolar growth appears relatively small when teeth are absent. For this reason, in children with severe hypodontia, the anterior mandible may probably represent the most suiTable site for implant placement (57).

For the restoration, overdentures were the most commonly used, either with ball attachments (14,15) or with a metal reinforcement structure (41,18,38). The teeth were usually made of acrylic-based resin, supported by mucosa, but retained by the implants. The main advantages, in addition to improved mastication, are that they facilitate care and maintenance at home, and can be removed after each meal.

In children with ED (Ectodermal Dysplasia), the retention of conventional complete dentures or removable skeletal prostheses is even scarcer and more complex due to their characteristics: partial or total absence of teeth, slight salivary secretion and thin alveolar bone (20,44). When analyzing the different studies, it has been observed that the placement of implants in patients with ED has a high predictability, with positive clinical results among which can be found the improvement of masticatory capacity and life quality as well as a phonetic improvement which cause increasing levels of self-esteem and social acceptability (34,20,51).

The heterogeneity of the treatments analyzed and the lack of unified protocols in terms of diagnosis and follow-up have made it difficult to assess the quality of the studies. Similarly, when defining the success rate by the different authors, there is a great diversity of criteria. Although the success of the implant is generally defined as an asymptomatic and functional implant, the definition varies depending on the studies analyzed. Brahim *et al*. consider success when there is a positive reaction of the soft and bone tissues surrounding the implant, and Sweeny *et al*., when the function is asymptomatic, absence of peri-implantitis, lack of bleeding on gentle probing, lack of suppuration, marginal inflammation and mobility. On the contrary, authors such as Bergendal and Lambert consider success to be the absence of complications during follow-up, without defining specific specifications, as is the case of Heureber *et al*., who provide a more complete exposition of the success criteria (value of peri-implant probing ≤5 mm, bleeding on probing (BoP) negative, bone loss <0.2 mm). Furthermore, the small sample size prevents experimental studies from being carried out.

It would be necessary to standardize the protocol for the planning, placement and follow-up of patients. This would make it possible to be more efficient and obtain better results. In diagnosis, the use of lateral radiography or teleradiography would be fundamental (32). It would allow the facial pattern to be recognized, growth to be evaluated and the changes produced during the maturation of tissues and bone structures to be determined (32).

The results obtained in this review should be interpreted with caution since most of the studies are clinical cases, or retrospective and of small sample size, with limited follow-up times. It seems reasonable to perform future studies with a higher level of evidence in order to establish surgical and patient follow-up recommendations.

Given the particular situation of pediatric patients with severe agenesis or ED, the prescription of dental implants for early insertion will be an alternative to be considered within the therapeutic possibilities.

Implants can improve the quality of life of children and adolescents with multiple agenesis, as long as the age, location, bone arrangement and psychological preparation of the child and family are taken into consideration.

Establishing a protocol with follow-up visits will allow a continuous evaluation, as well as an early detection of future complications. Informing parents or guardians of the therapeutic limitations, as well as reflect in the informed consent the lack of clinical predictability

## Conclusions

This review breaks the paradigm of implant placement at pediatric ages. Despite it has traditionally been a controversial topic, good results have been reported in the literature:

- Implants would be a valid option in cases of multiple agenesis in pediatric patients considering the age, location, bone disposition and the psychological preparation of the child and the family.

- Used with the appropriate precautions, these treatments can offer the child or adolescent a better aesthetics and function.

- Implants in children with multiple agenesis avoid a greater number of emergency visits and offer a better quality of life.
